# Perceived risk of tuberculosis infection among healthcare workers in Swaziland

**DOI:** 10.1186/s12879-016-2029-6

**Published:** 2016-11-23

**Authors:** Yi-Hao Weng, Patience Thulile Bhembe, Hung-Yi Chiou, Chun-Yuh Yang, Ya-Wen Chiu

**Affiliations:** 1 Department of Pediatrics, Chang Gung Memorial Hospital, Chang Gung University College of Medicine, Taipei, Taiwan; 2 Department of Nursing, Faculty of Health Sciences, Southern Africa Nazarene University, Manzini, Swaziland; 3 Master Program in Global Health and Development, College of Public Health, Taipei Medical University, 250 Wu-Hsing Street, Taipei, 110 Taiwan; 4 School of Public Health, College of Public Health, Taipei Medical University, Taipei, Taiwan; 5 Health Policy and Care Research Center, College of Public Health, Taipei Medical University, Taipei, Taiwan; 6 Department of Public Health, Kaohsiung Medical University, Kaohsiung, Taiwan

**Keywords:** Infection control, N95 respirator mask, Sputum, Tuberculosis, Swaziland

## Abstract

**Background:**

The incidence of tuberculosis (TB) in the Kingdom of Swaziland is extremely high. How healthcare workers (HCWs) in Swaziland perceive infection control (IC) measures for preventing TB transmission is unclear. This study aimed to determine perceived risk of TB infection in relation to IC measures among HCWs in three institutions of Swaziland.

**Methods:**

A cross-sectional questionnaire survey was conducted in 2014. Demographic data and IC measures were collected from main and allied HCWs.

**Results:**

In total, 186 HCWs (19 doctors, 99 nurses, and 68 allied HCWs) were enrolled. The multivariate logistic regression analyses revealed that nurses (OR = 39.87, 95% CI = 2.721–584.3) and other HCWs (OR =99.34, 95% CI = 7.469–1321) perceived a higher TB infection risk than did doctors. Moreover, HCWs working for <4 years at the TB department perceived a lower TB infection risk (OR = 0.099, 95% CI = 0.022–0.453). Availability of N95 respirator masks (OR = 0.055, 95% CI = 0.005–0.586) and a designated sputum collection area (OR = 0.142, 95% CI = 0.037–0.545) also carried lower TB infection risks.

**Conclusion:**

This study depicts the current status of IC measures for TB infection in a high prevalence country. The results suggest that HCWs perceived a greater TB infection risk at inadequate environmental IC measures.

**Electronic supplementary material:**

The online version of this article (doi:10.1186/s12879-016-2029-6) contains supplementary material, which is available to authorized users.

## Background

Tuberculosis (TB) constitutes an important proportion of the global disease burden. Nosocomial TB transmission has been well-documented worldwide, regardless of the local TB incidence, engendering a considerable risk of TB infection among healthcare workers (HCWs) [1–5]. Several interventions are helpful in preventing TB transmission to HCWs [6]. Administrative infection control (IC) measures, such as educational programs, can reduce TB infection [7]. In addition, environmental control and personal protective equipment are crucial contributors to the prevention of TB transmission [1, 8]. The compliance of HCWs with IC measures is influenced by organizational, environmental, and individual factors. However, whether the perception of TB transmission is associated with these factors is unclear. Because adherence to IC measures is mostly driven by HCWs’ belief in the effectiveness of these measures, understanding how HCWs perceive IC measures for preventing TB transmission is imperative.

The incidence of TB in the Kingdom of Swaziland is extremely high [9–12]. Therefore, Bacillus Calmette-Guerin (BCG) vaccinations are routinely administered and TB control programs have been established. However, how HCWs perceive IC measures for preventing TB transmission is unclear [9, 11]. In the current study, we used a questionnaire survey to investigate the perceived risk and IC measures of TB infection among HCWs of three health facilities in Swaziland. We further analyzed the perceived risk of TB infection in relation to IC measures. Our results can highlight the correlation between the perceived risk and IC measures of TB infection in a high-prevalence country.

## Methods

### Design and participants

A structured questionnaire was developed using data from published articles and the World Health Organization’s conceptual framework and TB IC guidelines [13–15]. This mixed-methods study was conducted during a 3-month period from July to September 2014.

We enrolled HCWs employed at the National TB Hospital, Raleigh Fitkin Memorial (RFM) Hospital, and Southern Africa Nazarene University (SANU) located in the Manzini Region, Swaziland. All HCWs in these three institutes were eligible for enrollment, including physicians, nurses, and allied HCWs. Workers not related with clinical health care were not included into this study. Those with age under 18 were not enrolled. The total number of eligible participants was obtained from the departments of human resources. A power calculation was not done beforehand.

The National TB Hospital is a 100-bed public hospital for the treatment of TB. The RFM Hospital is a 350-bed regional referral and teaching hospital providing daily services to approximately 500 patients in outpatient departments. SANU is a new university developed by combining three colleges, the Nazarene College of Nursing, Nazarene Teacher Training, and the Nazarene College of Theology. The study sites were classified into two categories: TB facility (National TB Hospital) and non-TB facility (RFM Hospital and SANU). TB facility was defined the referral hospital for TB and non-TB facility was defined as healthcare institute for general diseases.

### Questionnaire

The survey included items for measuring the profile of IC measures for preventing TB transmission including administrative measures, environmental control, and personal protective behavior (see Additional file 1). Questions related to the risk of TB infection were rated using a 5-point Likert scale (*strongly agree*, *agree*, *neutral*, *disagree*, and *strongly disagree*). In addition, demographic data including age, sex, profession, work period, educational level, and marital status were obtained. The questionnaires were sent to managers of the three institutions, and they were asked to distribute them to the participants. The questionnaire was completed by the participants themselves.

All questions were prepared in English and then translated into Swazi by a native speaker; subsequently, another translator fluent in both Swazi and English back-translated the questions from Swazi into English to ensure the accuracy of the translation.

### Validity and reliability

Content validity was examined by four experts. The specialties of the experts included global health, infectious diseases, survey methodology, statistics, epidemiology, and tropical medicine. The internal consistency of all indices was estimated using Cronbach’s coefficient alpha. In this survey, a content validity index of 0.86 and Cronbach’s coefficient alpha of 0.88 indicated adequate validity and reliability, respectively, of all parameters in the questionnaire.

### Ethical considerations

This study was approved by the Scientific and Ethics Committee of the Ministry of Health and Social Welfare in Swaziland. In addition, permissions were obtained from the three institutions. An introductory letter stating the study purpose and promising confidentiality was sent along with the questionnaire. Informed consent in written form was obtained from all the enrolled participants.

### Statistical analyses

A self-rating of *strongly agree* or *agree* was considered as a favorable answer (high risk), whereas a self-rating of *neutral*, *disagree*, or *strongly disagree* was considered as an unfavorable answer (low risk). Statistical analyses were conducted using a commercially available program (SPSS 19.0 for Windows, SPSS, Chicago, Il, USA). Categorical variables were analyzed using the chi-squared test or Fisher’s exact test when appropriate. A multivariate logistic regression model was used to adjust for possible confounders. Odds ratios and 95% confidence intervals were calculated after adjustments for control variables. Significance was defined as *P* < 0.05.

## Results

### Demographic data of the participants

Overall, 255 HCWs were eligible for enrollment. In total, 186 valid questionnaires were returned (97 HCWs from the National TB Hospital, 57 HCWs from the RFM Hospital, and 32 HCWs from SANU), indicating a return rate of 72.9%. In specific, the response rate was 84.3% in the National TB Hospital, 87.7% in the RFM Hospital, and 42.7% in SANU. The demographic information of all the participants is listed in Table 1. We enrolled 19 medical doctors, 99 nurses (4 sisters in charge, 81 nurses, and 14 nursing assistants), and 68 allied HCWs (32 students, 8 laboratory technicians, 15 ward aides, and 13 other HCWs).

**Table 1:** Demographic information of the participants (*n* = 186)

Demographic data	Number	Percent
Working place		
National TB Hospital	97	52.2
RFM Hospital	57	30.6
SANU	32	17.2
Age		
19–25	44	23.7
26–35	87	46.8
> 35	55	29.5
Sex		
Female	129	69.4
Male	57	30.6
Educational level		
Secondary school	6	3.2
High school	40	21.5
Diploma and post Diploma certificate	92	49.5
Degree^a^	48	25.8
Marital status		
Married	78	41.9
Single	91	48.9
Widowed	3	1.6
Divorced	14	7.6
Profession		
Nurse	99	53.2
Doctor	19	10.2
Allied HCW	68	36.6
Work period in the TB department		
< 4 years	121	65.0
4–9 years	58	31.2
> 9 years	7	3.8

^a^including bachelor, master, and PhD

The TB department was defined as departmental service for TB. Approximately two-third of participants worked in the TB department for less than 4 years.

### Infection control for TB

Table 2 lists IC measures taken in TB and non-TB facilities. Overall, the most common IC measure was individual behavior including wearing a protective gown when taking care of patients with TB (93.0%) and washing hands in between different patients’ care (80.1%). Among the environmental control measures, the most common measures were natural ventilation (evidence of unrestricted airflow, high ceiling >3 m, vents, and windows) (88.2%) and opening windows during working hours (84.9%). The least adopted IC measures were the availability of a designated area for sputum collection (26.3%) and air purification through ultraviolet germicidal irradiation or high-efficiency particulate air filtration (29.6%).

**Table 2:** Infection control (IC) for TB

Facility	Total	National TB Hospital	RFM Hospital + SANU	*P* value
	*N* = 186	*N* = 97	*N* = 89	
IC for TB	n (%)	n (%)	n (%)	
Administrative measures				
IC plan	100 (53.8)	40 (41.2)	60 (67.4)	<0.001
Routine IC training	77 (41.4)	30 (30.9)	47 (52.8)	0.002
Routine TB screen	95 (51.1)	78 (80.4)	17 (19.1)	<0.001
Environmental control				
Designated area for sputum collection	49 (26.3)	37 (38.1)	12 (13.5)	<0.001
Mechanical ventilation	90 (48.4)	81 (83.5)	9 (10.1)	<0.001
Natural ventilation	164 (88.2)	93 (95.9)	71 (79.8)	0.001
Window opening during working hours	158 (84.9)	70 (72.2)	88 (98.9)	<0.001
Air purification	55 (29.6)	54 (55.7)	1 (1.1)	<0.001
N95 respirator mask availability	101 (54.3)	81 (83.5)	20 (22.5)	<0.001
Detergent availability	77 (41.4)	51 (52.6)	26 (29.2)	0.001
Personal behavior				
Sputum induction/collection	76 (40.9)	43 (44.3)	33 (37.1)	0.315
Wearing of protective gowns	173 (93.0)	96 (99.0)	77 (86.5)	0.001
Washing of hands	149 (80.1)	75 (77.3)	74 (83.1)	0.320

The IC measures, including administrative measures, environmental control, and personal behaviors, significantly differed between the TB and non-TB facilities. IC planning, routine IC training, and window opening during working hours were more common in the non-TB facilities than in the TB facility. By contrast, routine TB screening, availability of a designated area for sputum collection, mechanical and natural ventilation, air cleaning, and access to N95 respirator masks and detergents were higher in the TB facility than in the non-TB facilities. Furthermore, compared with the non-TB facilities, the HCWs in the TB facility more often wore protective gowns when contacting patients.

### Perceived risk of TB infection

Overall, 143 participants (76.9%) rated that they were at risk for TB infection (Table 3). The perceived risk was lower in HCWs with the following demographic characteristics: (1) working in the National TB Hospital, (2) higher academic degree (diploma and degree), (3) men, (4) unmarried, (5) working in the TB department for <4 years, and (6) physicians and nurses. Moreover, the risk was lower in the presence of the following IC measures: (1) routine TB screening, (2) designated area for sputum collection, (3) N95 respirator mask availability, (4) detergent availability, and (5) wearing of protective gowns. By contrast, the risk was higher in the presence of an IC plan.

**Table 3:** Perceived risk of TB infection

Perceived risk, n (%)	High	Low	*P* value
	*N* = 143	*N* = 43	
Working place			0.003
National TB Hospital	66 (46.2)	31 (72.1)	
RFM Hospital + SANU	77 (53.8)	12 (27.9)	
Age			0.803
19–25	33 (23.1)	11 (25.6)	
26–35	66 (46.2)	21 (48.8)	
> 35	44 (30.7)	11 (25.6)	
Educational level			0.002
Secondary or High school	44 (30.8)	2 (4.7)	
Diploma and post Diploma certificate	63 (44.0)	29 (67.4)	
Degree	36 (25.2)	12 (27.9)	
Profession			<0.001
Nurse	71 (49.7)	28 (65.1)	
Doctor	10 (7.00)	9 (20.9)	
Allied HCW	62 (43.3)	6 (14.0)	
Working in the TB department for <4 years	85 (59.4)	36 (83.7)	0.003
Married	67 (46.9)	11 (25.6)	0.013
Male sex	37 (25.9)	20 (46.5)	0.010
Administrative measures			
IC plan	86 (60.1)	14 (32.6)	0.001
Routine IC training	64 (44.8)	13 (30.2)	0.090
Routine TB screening	63 (44.1)	32 (74.4)	<0.001
Environmental control			
Designated area for sputum collection	25 (17.5)	24 (55.8)	<0.001
Mechanical ventilation	64 (44.8)	26 (60.5)	0.071
Natural ventilation	123 (86.0)	41 (95.3)	0.097
Window opening during working hours	125 (87.4)	33 (76.7)	0.086
Air purification	41 (28.7)	14 (32.6)	0.624
N95 respirator mask availability	65 (45.5)	36 (83.7)	<0.001
Detergent availability	51 (35.7)	26 (60.5)	0.004
Personal behavior			
Sputum induction/collection	59 (41.3)	17 (39.5)	0.840
Wearing of protective gowns	130 (90.9)	43 (100)	0.041
Washing of hands	114 (79.7)	35 (81.4)	0.809

### Multivariate logistic regression model

Table 4 presents the results of the multivariate logistic regression analysis performed to adjust for possible confounders of the perceived TB infection risk. Wearing protective gowns was not included in the regression model because this group contained no participant. The following four characteristics were associated with the TB infection risk: two demographic characteristics (profession and work period in the TB department) and two environmental IC measures (designated area for sputum collection and N95 respirator mask availability). The TB infection risk was lower in medical doctors than in nurses and allied HCWs. In addition, the perceived TB infection risk was higher in HCWs with a work period of ≥4 years in the TB department than in those with a work period of <4 years in the TB department. With regard to environmental IC measures, the availability of N95 respirator masks and presence of a designated area for sputum collection were associated with a lower TB infection risk.

**Table 4:** Risk assessment for TB infection by the multivariate logistic regression model

	OR	95% CI	*P* value
Working place			
National TB Hospital	0.718	0.120–4.287	0.717
RFM Hospital + SANU	*reference*		
Age			
19–25	1.065	0.132–8.617	0.953
26–35	1.582	0.240–10.41	0.633
> 35	*reference*		
Profession			
Nurse	39.87	2.721–584.3	0.006
Doctor	*reference*		
Allied HCW	99.34	7.469–1321	<0.001
Educational level			
Secondary or High school	0.588	0.035–9.914	0.713
Diploma and post Diploma certificate	0.186	0.014–2.454	0.201
Degree	*reference*		
Working in the TB department for <4 years	0.099	0.022–0.453	0.003
Married	1.721	0.915–3.236	0.092
Male sex	0.333	0.103–1.071	0.065
Administrative measures			
IC plan	0.888	0.223–3.543	0.867
Routine IC training	0.982	0.310–3.113	0.976
Routine TB screening	0.774	0.111–5.404	0.796
Environmental control			
Designated area for sputum collection	0.142	0.037–0.545	0.004
Mechanical ventilation	0.981	0.165–5.848	0.983
Natural ventilation	1.546	0.525–4.548	0.429
Window opening during working hours	3.676	0.818–16.52	0.089
Air purification	1.647	0.984–2.757	0.058
N95 respirator mask availability	0.055	0.005–0.586	0.016
Detergent availability	2.053	0.614–6.867	0.243
Personal behavior			
Sputum induction/collection	0.750	0.177–3.175	0.696
Washing of hands	0.498	0.116–2.147	0.350

## Discussion

In this study, we investigated the perception of HCWs regarding IC measures for TB prevention in Swaziland. In addition, we evaluated the correlation of their perceived TB infection risk with IC measures. In our study, approximately half of the participants stated that administrative and environmental measures were not adhered to. In addition to the problems of building design, we observed that almost a half of HCWs reported a shortage of relevant equipment, including N95 respirator mask and detergent, in three health institutions of Swaziland. These results are in accordance with those of previous reports that have revealed failure in compliance with IC measures in resource-limited settings [13, 16–18]. There is evidence confirming that the TB infection risk appears to be particularly high in institutions with inadequate IC measures [14]. Environmental IC measures are considered the most effective methods for reducing the risk of TB infection. Our study results indicate that environmental IC measures are the most critical factors contributing to the perceived TB infection risk. These findings are consistent with those of previous studies [6, 7, 19]. Because of the high TB burden in Swaziland, ensuring compliance with required measures for TB prevention is imperative.

The availability of personal protective equipment and engineering control has been a critical factor related to safety in hospital environments [20]. In this study, we observed a correlation between the perceived TB risk and the availability of N95 respirator masks. N95 respirator masks provide a high filtration barrier and protect HCWs from TB infection. Therefore, it is recommended that HCWs should wear N95 respirator masks when taking care of patients with TB or those surmised to have TB [21]. However, adherence to the use of facial N95 respirator masks is not always observed in clinical practice because of their inadequate availability [8].

Safety in working environments has been a growing concern. However, quantitative methods investigating the perception of HCWs’ safety from TB infection is rare [22–24]. In our study, most HCWs believed that they were in an unsafe environment with a great risk of TB infection. The results are similar to those of a study conducted in Ethiopia that reported that 71% of HCWs perceived a high risk of occupational acquisition of TB [22]. We consider that the most likely reason is the failure of administrative and environmental IC measures. These measures are regarded as the most crucial defenses against TB infection. Thus, individual IC measures might not be effective when administrative and environmental measures are inadequate [18, 24].

Patients with TB are highly common in the National TB Hospital of Swaziland since this hospital is mainly responsible for the control of TB. Nevertheless, HCWs in this hospital perceived a low TB infection risk than those in other facilities did. This may be because environmental control measures and personal behaviors were more favorable in the National TB Hospital. Therefore, although HCWs in the National TB Hospital cared for patients with TB, they believed that they were safer.

Sputum collection is the standard practice for TB diagnosis [25]. Induced sputum can increase the microbiological diagnostic yield in patients surmised to have TB by stimulating sputum expectoration [26]. Sputum induction is a safe procedure for patients even in resource-poor settings [27]. During the procedure of sputum induction, protective measures are required for HCWs. In our study, HCWs involved in sputum induction and collection did not perceive a higher TB infection risk, suggesting that they believed that their protective measures were adequate.

In our study, the univariate analysis showed HCWs with IC plan perceived more risk of TB infection than those without IC plan. However, there was no significant difference by the multivariate logistic regression model. Therefore, we speculate that, despite of the presence of IC plan, HCWs perceived greater risk of TB infection when the environmental control was inadequate.

Our study has limitations that should be addressed. First, this was a self-administered survey and not an audit of actual practice. Hence, a discrepancy between self-reported and actual IC measures may be present [17]. However, our study design could accurately reflect the perceived risk. Second, the perceived risk is not equal to the real risk of TB infection. The risk of TB infection between HCWs and general population in Swaziland has not been determined yet. Nevertheless, healthcare quality could be influenced if HCWs perceived a high TB infection risk [19]. Third, our study did not investigate the correlation of HIV with TB infection. Further studies are needed to identify the perceived risk of TB infection among HIV-positive HCWs.

## Conclusions

Our study has clinical implications for strategy development to prevent TB infection of HCWs. The findings provide crucial information on the current status of IC measures for TB infection in Swaziland. We have identified HCWs perceived a greater TB infection risk with inadequate environmental IC measures. A significant correlation between environmental IC measures and the perceived TB infection risk was observed. HCWs perceived a high TB infection risk owing to the inadequate implementation of environmental IC measures. To maintain a safe workplace for HCWs, improving IC measures should be a top priority in Swaziland. First, N95 respirator masks should be available for all HCWs with risk of TB infection. Second, a designated area for sputum collection should be mandatory in order to decrease the TB infection of HCWs.

To our knowledge, this study is the first survey to assess the perceived risk of TB infection among HCWs in Swaziland. This study verified the current status of IC measures for TB infection in Swaziland. A majority of HCWs in Swaziland perceived risk of TB infection. Environmental IC measures were regarded as the most important factor of TB infection. Thus, ensuring compliance with TB prevention measures is important to reduce the risk of nosocomial TB infection among HCWs in Swaziland.

## Additional file

Additional file 1: Questionnaire in English. (PDF 223 kb)

## Abbreviations

HCWs: Healthcare workers
IC: Infection control
RFM Hospital: Raleigh Fitkin Memorial Hospital
SANU: Southern Africa Nazarene University
TB: Tuberculosis
