# Supplementary material for: Perceived risk of tuberculosis infection among healthcare workers in Swaziland
Source: BMC Infect Dis. 2016 Nov 23;16:697. doi: 10.1186/s12879-016-2029-6 (PMC5122014; doi:10.1186/s12879-016-2029-6)
Supplement: Additional file 1: — Questionnaire in English. (PDF 223 kb) [file 12879_2016_2029_MOESM1_ESM.pdf]

Please fill the questionnaire and tick where applicable.

Demographic data

1. What is your age? ....

2. What is your gender?

- ☐ Female
- ☐ Male

3. What is your working position in this facility?

- ☐ Sister in charge
- ☐ Medical doctor
- ☐ Nurse
- ☐ Nursing assistant
- ☐ Student nurse
- ☐ Laboratory technician
- ☐ Ward aides
- ☐ Other, specify ....

4. Education attainment

- ☐ Primary
- ☐ Secondary
- ☐ High school
- ☐ Diploma
- ☐ Post Diploma Certificate
- ☐ Bachelor's Degree
- ☐ Master's Degree
- ☐ PhD

5. Marital status

- ☐ Married
- ☐ Single

☐ Widowed

☐ Divorced

☐ On Separation

6. How long have you been working in this TB department?
   Years........................Months.......................................

Administrative control questions

7. Is the infection control plan of TB transmission to health workers from patients available in your department in writing?

☐ No

☐ Yes

8. Is there an ongoing training for healthcare workers about TB infection control practices?

☐ No

☐ Yes

9. Does your facility provide routine TB screening for the healthcare workers?

☐ No

☐ Yes, how often is it done?

  ☐ Every 6 months

  ☐ Once a year

  ☐ Every after two years

Environmental control questions

10. Describe the natural ventilation in your department (tick all that apply)

☐ Open windows on opposite wall, unrestricted flow

☐ High ceiling (>3meters)

☐ Vents and windows

☐ No windows

☐ Others

11. When do you keep windows open? (Tick all that apply)

☐ During the day

☐ At night

☐ During cold weather

☐ During hot weather

☐ Not open anytime

☐ Not applicable

12. Is there any mechanical ventilation in your department?

☐ Yes

☐ No

13. What air cleaning methods are used in the facility? (Tick all that apply)

☐ Ultra-violet germicidal irradiation

☐ HEPA (high efficiency particulate air) filtration

☐ Others (specify) ……………………………………………………

☐ None

14. Does your facility have a designated area away from other patients and workers where patients can produce sputum specimen?

☐ Yes

☐ No

15. Are the N95 masks always available?

☐ Yes

☐ No

16. Are the detergents for washing hands always available in your facility?

☐ Yes

☐ No

Personal respiratory control questions

17. Are you involved in sputum induction/collection procedures?

☐ Yes

☐ No

18. Do you wear any protective gown when you are taking care of patients with TB?

☐ Yes

☐ No

19. Do you always wash your hands in between different patients' care?

☐ Yes

☐ No

20. Do you believe you are at risk of developing active TB infection in your department?

☐ Strongly agree

☐ Agree

☐ Neutral

☐ Disagree

☐ Strongly disagree

Thank you for your time in answering the questions
